# Evaluating the Diabetes–Cardiology interface: a glimpse into the diabetes management of cardiology inpatients in western Sydney’s ‘diabetes hotspot’ and the establishment of a novel model of care

**DOI:** 10.1186/s13098-018-0393-7

**Published:** 2018-12-14

**Authors:** Ramy H. Bishay, Gideon Meyerowitz-Katz, David Chandrakumar, Rajini Jayaballa, Tien-Ming Hng, Mark Mclean, Dilini Punchihewa, Maiyoori Jeyaprakash, David Burgess, John Riskallah, Glen F. Maberly

**Affiliations:** 1 Western Sydney Diabetes, Integrated and Community Health Directorate, Department of Endocrinology and Diabetes, Blacktown Hospital, Western Sydney Local Health District, Sydney, NSW Australia; 2 School of Medicine, Western Sydney University, Sydney, NSW Australia; 3 School of Medicine, University of Sydney, Sydney, NSW Australia; 4 Department of Cardiology, Blacktown Hospital, Blacktown, NSW Australia; 5 Metabolic and Weight Loss Program, Blacktown Hospital, Western Sydney Local Health District, Sydney, NSW Australia; 6 Department of Endocrinology, Clinical School Building, Level 3, Western Sydney University Blacktown Campus, Blacktown Hospital, Marcel Crescent, Blacktown, NSW 2148 Australia

**Keywords:** In-patient diabetes, Health care delivery, Hypoglycaemia, SGLT-2 inhibitors, Incretin therapies, GLP-1 receptor analogues, Cardiovascular disease, Obesity, Cardiovascular mortality, Ischaemic heart disease

## Abstract

**Background:**

Approximately two-thirds of individuals presenting to emergency departments in Western Sydney have glucose dysregulation, accelerating their risk of cardiovascular disease (CVD). We evaluated the prevalence and management of type 2 diabetes (T2D) in cardiology inpatients in Western Sydney. A novel model of care between diabetes and cardiology specialist hospital teams (joint specialist case conferencing, JSCC) is described herein and aimed at aligning clinical services and upskilling both teams in the management of the cardiology inpatient with comorbid T2D.

**Methods:**

Cardiology inpatients at Blacktown-Mount Druitt Hospital were audited during a 1-month period.

**Results:**

233 patients were included, mean age 64 ± 16 years, 60% were male, 27% overweight and 35% obese. Known T2D comprised 36% (n = 84), whereas 6% (n = 15) had a new diagnosis of T2D, of which none of the latter were referred for inpatient/outpatient diabetes review. Approximately, 27% (n = 23) and 7% (n = 6) of known diabetes patients suffered hyper- and hypoglycaemia, respectively, and 51% (n = 43) had sub-optimally controlled T2D (i.e. HbA1c > 7.0%); over half (51%, n = 51) had coronary artery disease. Only two patients were treated with an SGLT2 inhibitor and no patients were on glucagon like peptide-1 receptor analogues. The majority were managed with metformin (62%) and therapies with high hypoglycaemic potential (e.g., sulfonylureas (29%)) and in those patients treated with insulin, premixed insulin was used in the majority of cases (47%).

**Conclusions:**

Undiagnosed T2D is prevalent and neglected in cardiology inpatients. Few patients with comorbid T2D and CVD were managed with therapies of proven cardiac and mortality benefit. Novel models of care may be beneficial in this high-risk group of patients and discussed herein is the establishment of the diabetes-cardiology JSCC service delivery model which has been established at our institution.

## Background

Given that people with type 2 diabetes mellitus (T2D) are more than 2–3 times more likely to die of cardiovascular disease (CVD) than people without T2D [1, 2], treatment paradigms have favourably shifted from a ‘glucose lowering’ to a ‘cardioprotective’ model of care [3–5]. Recent cardiovascular safety and non-inferiority trials of newer diabetes therapies show cardiovascular neutrality with dipeptidyl peptidase-4 inhibitors (DPP4i [e.g., sitagliptin]) [6] however, there was demonstrable reduction in CV mortality and hospitalisation for heart failure (HF) with sodium-glucose co-transporter 2 inhibitors (SGLT2i [e.g., empagliflozin, canagliflozin, dapagliflozin]) [7–9], and CV events with glucagon-like peptide 1 receptor agonists (GLP1-RA [e.g., liraglutide, semaglutide]) [10, 11]; this is in addition to added benefits of modest weight loss and improvement in blood pressure also seen with these agents.

Considering the robust clinical evidence, the uptake of ‘cardioprotective therapies’ may not be as sweeping as once thought [12], especially in primary care. These reasons include the lack of clinical inertia, the physician’s unfamiliarity with the newer therapies and of monitoring mechanisms, cost, side-effect profile [13], and perceived invasiveness of the injectable GLP1-RA. Prescribing trends in the UK still favoured metformin as the first-line treatment for T2D among primary care physicians (83.6%) however, sulphonylureas remained the second most common drug (41.4%), [14] despite the 10–17% increase in CV mortality, events, and hypoglycaemia [3]; in contrast, GLP-1 RA were rarely prescribed first-line or after metformin and DPP4i were prescribed at a mediocre 15.4%. Insulin prescriptions remained stable at 20–24% [14]. There is unanimous agreement regarding reducing the risk of hypoglycaemia in modern diabetes management, yet the same therapies (i.e., sulphonylureas, insulins) were being used widely in the community.

Western Sydney is recognised as having one of the most metabolically abnormal populations in Australia, with nearly two-thirds of all people being tested in tertiary hospital emergency departments having some form of glycaemic abnormality (38.4% with T2D, 27.4% with prediabetes) using glycosylated haemoglobin (HbA1c) measurements taken as part of a screening project [15]. Given the emphasis on CV outcomes with newer pharmacotherapies, this study focussed on admitted patients to the cardiology service. We hypothesised that the considerable metabolic burden of disease known in our region, would lead us to identify a large gap between evidence-based clinical management and real-world findings. Our aims were to evaluate the prevalence of metabolic disease in this cohort, audit the clinical and financial outcomes, and evaluate pharmacologic burden in those with T2D. Importantly, we endeavoured to capture the potential opportunities that exist in improving diabetes management in this high-risk group, with a view towards enhancing the collaboration between diabetes and cardiology services through joint specialist case conferencing (JSCC), a novel model of care that has been recently implemented in our unit (see “Discussion” section).

## Methods

### Electronic medical records

A 1-month retrospective audit was undertaken of all patients admitted under the Cardiology Service through the Emergency Department in the month of September 2016 in Blacktown Mt Druitt Hospital, Western Sydney Local Health District (New South Wales, Australia). The hospital uses electronic medical records for documentation such that all progress notes, medications, patient information and results of investigations (pathology and radiology) are collected and accessible via a secure user defined, login process.

### Data collection

Data collected was de-identified and included basic demographic information, weight (overweight, body mass index [BMI] 25–29.9 kg/m^2^; obese, BMI ≥ 30 kg/m^2^) and as having either incident or known prediabetes (HbA1c 39 to 46 mmol/mol, 5.7–6.4%) or T2D (HbA1c ≥ 48 mmol/mol or 6.5%) based on established definitions. Duration of diagnosis, diabetes treatment and the presence of micro- (retinopathy, neuropathy, nephropathy) and macrovascular (CV disease, peripheral vascular, cerebrovascular disease) complications were also obtained.

Primary reasons for admission, presence of coronary artery disease (CAD), HF, left ventricular ejection fraction (LVEF), valvular disease, the presence of other comorbid cardiopulmonary disease, risk factors, medications and laboratory data were obtained from the history or investigations (e.g., angiogram, echocardiogram). Cost of the inpatient stay was determined using national weighted activity unit (NWAU), a nationally standardised costing of health service activity, as well as the presence of hyper- or hypoglycaemia. Engagement with the diabetes service (referrals to diabetes educator and/or endocrinology registrar) were also documented to assess service utilization. This study was approved by the local human research committee.

### Exclusions

We excluded individuals with Type 1 diabetes, admissions for elective procedures (e.g., elective angiograms or percutaneous coronary interventions/angioplasty, DC cardioversion for atrial fibrillation/flutter, to perform transthoracic echocardiograms, awaiting preparation for coronary artery bypass graft procedure) readmissions within the audit month, incorrect admissions under cardiology and individuals not admitted through the emergency department. Admissions for elective procedures were defined using hospital coding as any admission that had a code of “elective”, no specific procedures were identified in the audit.

### Statistical analysis

All statistical analysis was conducted using SPSS software. Continuous variables were compared using a standard t-test, and categorical variables using Chi squared methodology. Bonferroni’s method was used to control for multiple comparisons, with p-values presented after correction.

## Results

Identified patients included 311 of which 235 were included in the analysis (51 excluded due to elective procedures, 25 when the exclusion criteria was applied); 136 were non-T2D and 99 had T2D (Table 1), indicating a prevalence of 42% for T2D. Study participants with T2D were more likely to be older, more overweight or obese and have a longer length of stay by nearly 2 days (43 h); gender distribution among T2D cohort was equal. The T2D cohort were almost four times more likely to present with IHD (mostly ST segment elevation myocardial infarction, STEMI) and nearly twice the rates of heart failure then the non-diabetic group (all p < 0.0001; Table 1). The NWAU also appeared to be higher in the T2D versus non-T2D cohort, although this was not statistically significant (*p* = 0.07).

**Table 1:** Comparison of demographic, clinical and admission characteristics between study participants in those with and without type 2 diabetes mellitus

Parameter	Non-T2D	T2D	*P*-value
*N*	136	99	
Male (*N, %)*	85 (63%)	54 (54%)	NS
Mean age (years)	62 ± 17	67 ± 13	< 0.01*
Country of birth			
Australia/NA	88	84	NS
Europe	17	19	NS
Asia	10	15	NS
Middle East	6	5	NS
India	6	4	NS
Pacific Islands	5	6	NS
Aboriginal/Torres Strait Islander	3	1	NS
New Zealand	3	1	NS
Africa	1	1	NS
Overweight (BMI 25–29.9 kg/m^2^) (*N*)**	41**	21**	0.001*
Obese (BMI ≥ 30 kg/m^2^) (*N*)**	37**	45**	0.01*
Pre-diabetes (*N*; %)	72 (53)	–	–
Body mass index (kg/m^2^)	28 ± 5	32 ± 8	< 0.001*
Glycosylated haemoglobin (HbA1c)			
mmol/mol	39 ± 3	55 ± 9	< 0.001*
%	5.7 ± 0.4	7.3 ± 1.3	
Admission details			
Primary reason for admission			
Chest pain	35 (26)	28 (29)	< 0.0001***
Ischaemic heart disease	7 (5)	21 (21)	
Heart failure	13 (10)	18 (18)	
Arrhythmias	35 (26)	9 (9)	
Other heart disease	24 (18)	11 (11)	
Other (deemed non-cardiac)	21 (15)	12 (12)	
Length of stay (hours)	101 ± 130	144 ± 140	0.01*
National weighted activity unit (NWAU)	1.35 ± 1.52	1.72 ± 1.46	0.07

Values are shown as N (%) and mean ± SD*; T2D,* type 2 diabetes mellitus. *denotes statistical significance at *P* value < 0.05; **indicates some data missing or not recorded; ***denotes significant differences in distribution using Chi square test (*P* value < 0.05). Values in parentheses indicate percentage of the cohort; *NS* not statistically significant

Despite half of the non-T2D population having prediabetes based on screening HbA1c, participants with T2D had higher prevalence of CAD (p = 0.024), hypertension (p < 0.001), and dyslipidaemia (p = 0.002) but similar rates of stroke, previous PCI, HF and previous smoking (all p > 0.05; Table 2). There was higher pharmacologic burden in T2D patients (p = 0.002), with higher use of aspirin, angiotensin II receptor blockers, and loop diuretics, although likely due to the small subgroup sample size none of these values were statistically significant (p > 0.05). Of note, there was low uptake of ACEi or ARB in patients with T2D comorbid HF (39%, data not shown).

**Table 2:** Comparison of cardiac history, risk factors and pharmacotherapy between study participants in those with and without type 2 diabetes mellitus

Parameter	Non-T2D	T2D	*P*-value
*N*	136	99	
Coronary artery disease (*N*; %)	50 (37)	51 (51)	0.024*
Stroke (*N*; %)	8 (6)	12 (12)	NS
Previous coronary revascularisation	33 (24)	32 (32)	
Percutaneous coronary intervention^#^	24 (18)	23 (23)	
Coronary artery graft surgery	12 (9)	14 (14)	
Heart failure	42 (31)	33 (33)	
Left ventricle ejection fraction ≤ 40%	51 ± 18	53.6 ± 15.6	
Previous arrhythmia	49 (36)	26 (26)	
Valvular disease	51 (38)	36 (36)	
Cardiopulmonary disease and other risk factors			
Current smoker (*N*; %)	23 (17)	14 (14)	NS
Ex-smoker (*N*; %)	36 (26)	32 (32)	
Pulmonary hypertension	18 (13)	18 (18)	
Hypertension	80 (59)	80 (80)	< 0.001*
Dyslipidaemia	56 (41)	61 (61)	0.002* NS
Total cholesterol (mmol/L)	4.3 ± 1.3	4.0 ± 1.4	
LDL-c (mmol/L)	2.4 ± 1.1	2.2 ± 1.9	
HDL-c (mmol/L)	1.1 ± 0.2	0.9 ± 1.1	
Triglycerides (mmol/L)	1.7 ± 0.8	3.3 ± 2.1	
Chronic obstructive pulmonary disease	9 (7)	10 (10)	NS
Asthma	18 (13)	14 (14)	NS
Cardiac medications			
Aspirin	38 (28)	50 (50)	0.002*
Dual anti-platelet agents	10 (7)	16 (16)	NS
Clopidogrel	13 (10)	18 (18)	
Prasugrel	1 (0.7)	2 (2)	
Ticagrelor	2 (1)	2 (2)	
Warfarin	5 (4)	5 (5)	
Non-vitamin K antagonist oral anticoagulants	18 (13)	9 (9)	
Nitrates	9 (7)	10 (10)	
Ivabradine	2 (1)	3 (3)	
Digoxin	16 (12)	5 (5)	
Beta-blockers	44 (32)	44 (44)	
Alpha-blockers	9 (7)	9 (9)	
Calcium channel blocker	22 (16)	22 (22)	
Angiotensin converting enzyme inhibitors	31 (23)	19 (19)	
Angiotensin II receptor blockers	31 (23)	44 (44)	0.002*
Thiazide diuretic	9 (7)	12 (12)	NS
Loop diuretic	21 (15)	32 (32)	0.007*
Aldosterone receptor blocker	10 (7)	11 (11)	NS
Centrally-acting antihypertensive	1 (< 1)	1(1)	< 0.001*
Statins	61 (45)	73 (74)	NS
Ezetimibe	8 (6)	12 (12)	

Values are shown as N (%) and mean ± SD*; LDL*-*c* low density lipoprotein cholesterol, *HDL*-*c* high density lipoprotein cholesterol. *denotes statistical significance at *P* value < 0.05; **indicates some data missing or not recorded; ***denotes significant differences in distribution using Chi square test (*P* value < 0.05); ^#^T2D cohort—PCI revealed disease in 17 involving left anterior descending artery, 15 left circumflex, 14 right coronary artery, and 4 left main. Values in parentheses indicate percentage of the cohort; *NS* not statistically significant

Incident T2D cases were found in 14 patients (Table 3), of whom none were referred to the diabetes service or recommended for either general physician or specialist review on discharge; only a fraction had the diagnosis mentioned in their discharge letter (21%) or coded appropriately as a new diagnosis in the medical record (36%). Morbidity in the overall T2D cohort was high with micro- and macrovascular complications present in 40% and 63%, respectively. Referral to the diabetes service was low (9% of prevalent and 0% in incident cases). Most patients were managed with metformin and/or sulphonylureas being the second most common therapy prescribed, whereas SGLT2i and GLP1-RA were rarely prescribed and insulin use was dominated by premixed preparation (47%) with an out-of-target mean HbA1c (68 mmol/mol or 8.4%).

**Table 3:** Characteristics and management of study participants with type 2 diabetes mellitus

Parameter	N = 99
Incident T2D	14 (14%)
Referred for inpatient diabetes review	0/14
New diagnosis mentioned in discharge letter	3/14 (21%)
New diagnosis coded for in medical record	5/14 (36%)
Recommended for endocrinology review after discharge	0/14
Known T2D	85 (85%)
Referred for inpatient diabetes review	9/85 (11%)
Diagnosis mentioned in discharge letter	82/85 (96%)
Diagnosis coded for in medical record	82/85 (96%)
Recommended for endocrinology review after discharge	11/85 (13%)
Management of known T2D	85 (85%)
Hyperglycaemia	23/85 (27%)
Hypoglycaemia	6/85 (7%)
HbA1c > 7% (> 53 mmol/mol)	43/85 (56%)
Microvascular complications	40 (40%)
Peripheral neuropathy	7 (7%)
Retinopathy	5 (5%)
Nephropathy (2 on dialysis)	46 (46%)
Macrovascular complications	63 (63%)
Coronary artery disease	51 (51%)
Ischaemic heart disease	53 (53%)
Peripheral vascular disease	6 (6%)
Carotid stenosis	4 (4%)
Stroke/transient ischaemic attack	12 (12%)
Diabetes treatment	
Metformin	61 (62%)
Sulphonylurea	29 (29%)
Dipeptidyl peptidase-4 inhibitor	13 (13%)
Sodium glucose co-transporter 2 inhibitor	2 (2%)
Glucagon-like peptide-1 receptor analogue	0
Acarbose	0
Thiazolidinediones	0
None	26 (26%)
Insulin	17 (17%)
Mean total daily dose (U)	77.7 ± 53
Basal (mean HbA1c 78 mmol/mol, 9.3%)	4/17 (24%)
Premixed (mean HbA1c 68 mmol/mol, 8.4%)	8/17 (47%)
Basal-bolus (mean HbA1c 56 mmol/mol, 7.3%)	5/17 (29%)

Very few patients had an accurate documentation of duration of diagnosis of T2D and therefore this was not included. In total, 6 patients had documented hypoglycaemia (BGL < 4.0 mmol/L) of whom all were on either insulin or sulphonylurea, and 35 experienced hyperglycaemia (BGL ≥ 14 mmol/L) during their hospital admission.

## Discussion

Nearly 70% of people with T2D will die of CVD [16] therefore, focussing efforts to intensify case detection and enhance diabetes management to positively affect the long-term quality of life and survival in T2D patients is a major health priority.

Our audit revealed that people with T2D made up nearly 2 in 5 cardiology inpatients (14% were new diagnoses) and that T2D participants were unsurprisingly more obese or overweight, have comorbid CAD, stroke and as well as higher pharmacologic use than the non-T2D cohort. They were also more likely to present with heart failure (10 vs 18%, p < 0.0001, Table 1). In community-dwelling older adults (65–74 years—similar to our study mean age), national data reveals that among those with CVD, T2D was comorbid in 19.4%; conversely, those with T2D, CAD was present in 10.6%, HF in 8.2% and cerebrovascular disease in 2.4% [17]. In contrast, our study sample had nearly 5×, 4× and 5× the rates of CAD (51%), HF (33%) and cerebrovascular disease (stroke or TIA, 12%), suggesting the presence of increased disease burden due to hospital bias of admitted patients. We found that none of the incident and only 9% of prevalent T2D cases were referred for inpatient review (11% for outpatient review), illustrating the need to increase communication between the cardiology and diabetes services to better deliver care in this high-risk group.

Our study revealed that 47% of insulin-requiring patients were on premixed insulin, and that after metformin, sulphonylureas were the most commonly prescribed oral agent (29%). Both therapies are more likely to be associated with hypoglycaemia and weight gain, and adversely affect individuals with CVD, particularly given the association of hypoglycaemia with arrhythmia and sudden death [3, 18, 19], as well as the blunted hypoglycaemic awareness in the context of beta-blocker use in these patients.

In addition to positively improving glycaemic control, many of the ‘newer’ diabetes therapies (i.e., SGLT2i, GLP1-RA, DPP4i) have extra-glycaemic benefits, such as reduction in LDL-c, improvements in HDL-c, stabilisation of endothelial cells, reduction in inflammation, regulating adipokine release with the added benefits of weight loss [20]. The low uptake of these drugs suggests that these therapies are not being appropriately considered locally in CVD patients with T2D. In fact, the Food and Drug Administration added a cardiovascular mortality indication in 2016 for empagliflozin, and both semaglutide and liraglutide have shown CV benefits as noted previously [10, 11]. Consequently, national and international guidelines should give consideration to escalating these new therapies as second line treatment in preference over the use of sulphonylureas (and provide clear contraindications for the use of thiazolidinediones) for people with T2D and CVD.

We commenced this audit to establish a more comprehensive approach to the problem of CV morbidity and mortality in T2D patients, specifically with the use of a novel integrated model of care using JSCC. Joint Specialist Case Conferencing comprises of an integrated discussion about a patients’ diabetes management between the diabetes specialist, resident, diabetes educator, patient and primary care physician and is carried out in the primary care clinic for 30-min consultations per patient. We have previously shown that JSCC lead to benefits after a single visit to primary care physicians, including an absolute reduction of − 0.87% in HbA1c and reductions in blood pressure, lipid profile and weight, and that the results were durable to 3-years [21].

**Fig. 1:**
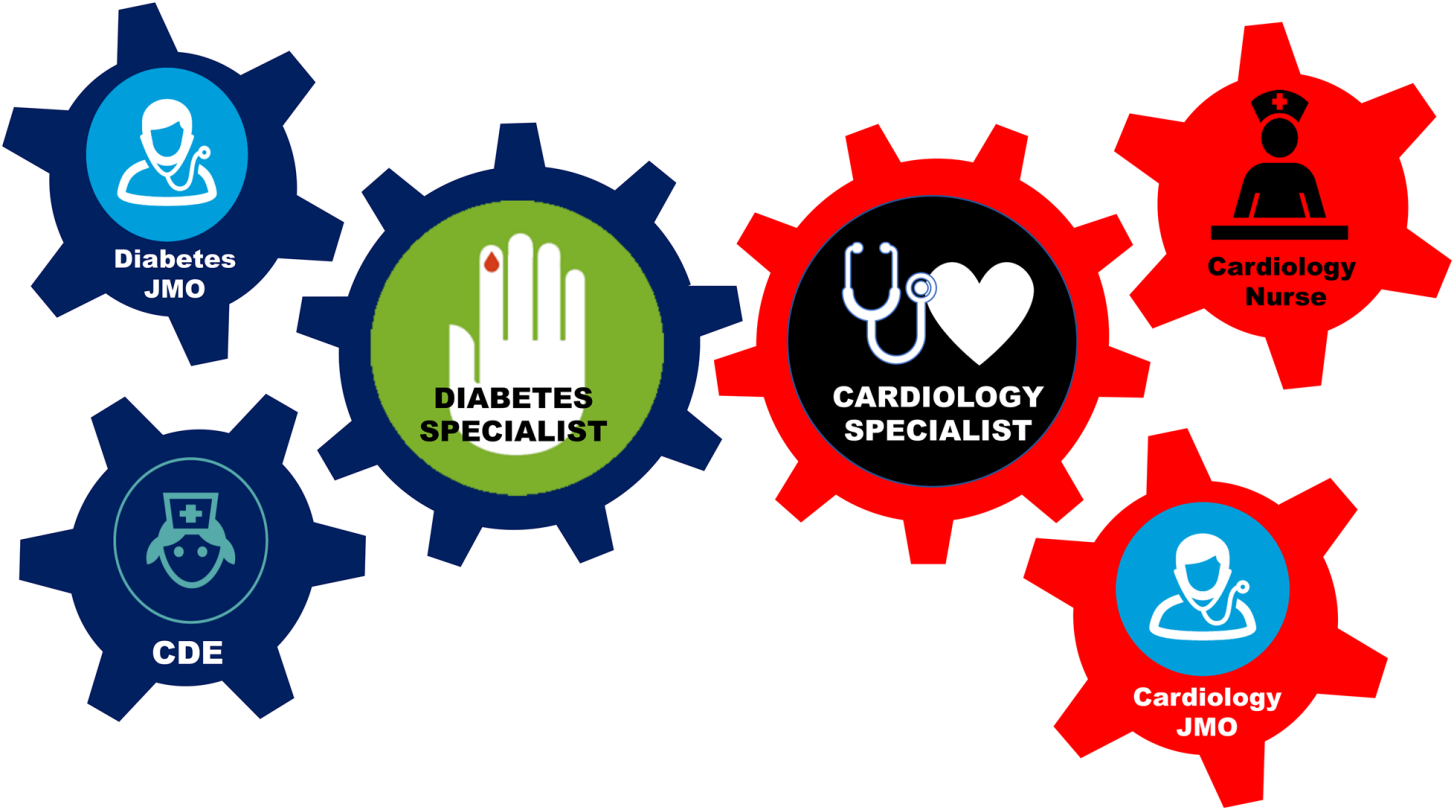
Schema of joint specialist case conferencing (JSCC) between diabetes and cardiology specialist teams. A novel model of care between both inpatient hospital teams to align, upskill and enhance hospital management of the cardiology inpatient with diabetes. *JMO* junior medical officer, *CDE* certified diabetes educator

Given that many cardiology inpatient services are primarily focused on the immediate management of acute and subacute emergencies, chronic conditions such as T2D may be overlooked. As such, we have similarly instituted JSCC with our cardiology hospital specialist team on a monthly basis in a modified format (i.e., patient not present but discussed in a round table setting with clinicians and diabetes educators and cardiology nurses present, see Fig. 1), with a focus on building capacity within the cardiology specialist team to better manage diabetes, with added benefits of increasing communication and collaboration between the departments. A protocol for the management of diabetes with a focus on cardiovascular outcomes is also being developed. A second follow-up audit is planned to assess the impact of this intervention in an age- and gender-matched cohort. Limitations of the study include its retrospective nature, which is inherent in audit-type studies, as well as lack of mortality data; follow-up studies are in progress.

## Conclusions

Altogether, this audit reveals the high prevalence of T2D in one of the most metabolically challenging, populations in Australia where there is a high burden of concurrent CVD. Greater collaboration should be sought in all tertiary hospitals where both cardiology and diabetes teams co-exist and a format similar to the JSCC model described here be considered to strengthen the diabetes-cardiology clinical service interface. Cardioprotective diabetes therapies have a unique niche in the CV patient with concomitant T2D and should guide appropriate management to reduce mortality and morbidity in this high-risk group.

## Abbreviations

ACEi: angiotensin converting enzyme inhibitor
ARB: angiotensin II receptor blocker
BGL: blood glucose level
BMI: body mass index
CAD: coronary artery disease
CAGS: coronary artery bypass graft surgery
CDE: certified diabetes educator
COPD: chronic obstructive pulmonary disease
CV: cardiovascular
CVD: cardiovascular disease
DAPT: dual antiplatelet therapy
DPP4i: dipeptidyl peptidase-4 inhibitors
GLP1-RA: glucagon-like peptide-1 receptor analogues
HbA1c: glycosylated haemoglobin
HDL-c: high density lipoprotein cholesterol
HF: heart failure
HTN: hypertension
IHD: ischaemic heart disease
ISMN: isosorbide mononitrate
JMO: junior medical officer
JSCC: joint specialist case conferencing
LDL-c: low density lipoprotein cholesterol
LOS: length of stay
LVEF: left ventricular ejection fraction
NOAC: non-vitamin K antagonist oral anticoagulants
NWAU: national weighted activity unit
PCI: percutaneous coronary intervention
PVD: peripheral vascular disease
SGLT2i: sodium glucose co-transporter 2 receptor inhibitors
STEMI: ST segment elevation myocardial infarction
T2D: type 2 diabetes mellitus
TIA: transient ischaemic attack
